# Risk factor analysis for a rapid progression of chronic kidney disease

**DOI:** 10.1093/ndt/gfad271

**Published:** 2024-01-02

**Authors:** Anne H S Vestergaard, Simon K Jensen, Uffe Heide-Jørgensen, Line E Frederiksen, Henrik Birn, Dorte E Jarbøl, Jens Søndergaard, Frederik Persson, Reimar W Thomsen, Christian F Christiansen

**Affiliations:** Department of Clinical Epidemiology, Department of Clinical Medicine, Aarhus University and Aarhus University Hospital, Aarhus, Denmark; Department of Clinical Epidemiology, Department of Clinical Medicine, Aarhus University and Aarhus University Hospital, Aarhus, Denmark; Department of Clinical Epidemiology, Department of Clinical Medicine, Aarhus University and Aarhus University Hospital, Aarhus, Denmark; Cardiovascular, Renal and Metabolism, Medical Department, BioPharmaceuticals, AstraZeneca, Copenhagen, Denmark; Departments of Clinical Medicine and Biomedicine, Aarhus University, Aarhus, Denmark; Department of Renal Medicine, Aarhus University Hospital, Aarhus, Denmark; Research Unit of General Practice, Department of Public Health, University of Southern Denmark, Odense, Denmark; Research Unit of General Practice, Department of Public Health, University of Southern Denmark, Odense, Denmark; Steno Diabetes Center Copenhagen, Herlev, Denmark; Department of Clinical Epidemiology, Department of Clinical Medicine, Aarhus University and Aarhus University Hospital, Aarhus, Denmark; Department of Clinical Epidemiology, Department of Clinical Medicine, Aarhus University and Aarhus University Hospital, Aarhus, Denmark

**Keywords:** disease progression, hospitalization, mortality, renal insufficiency, risk

## Abstract

**Background:**

Chronic kidney disease (CKD) is a growing global health concern. Identifying individuals in routine clinical care with new-onset CKD at high risk of rapid progression of the disease is imperative to guide allocation of prophylactic interventions, but community-based data are limited. We aimed to examine the risk of rapid progression, kidney failure, hospitalization and death among adults with incident CKD stage G3 and to clarify the association between predefined risk markers and rapid CKD progression.

**Methods:**

Using plasma creatinine measurements for the entire Danish population from both hospitals and primary care, we conducted a nationwide, population-based cohort study, including adults in Denmark with incident CKD stage G3 in 2017–2020. We estimated 3-year risks of rapid progression (defined by a confirmed decline in estimated glomerular filtration rate of ≥5 mL/min/1.73 m^2^/year), kidney failure, all-cause hospitalization and death. To examine risk markers, we constructed a heat map showing the risk of rapid progression based on predefined markers: albuminuria, sex, diabetes and hypertension/cardiovascular disease.

**Results:**

Among 133 443 individuals with incident CKD stage G3, the 3-year risk of rapid progression was 14.6% [95% confidence interval (CI) 14.4–14.8]. The 3-year risks of kidney failure, hospitalization and death were 0.3% (95% CI 0.3–0.4), 53.3% (95% CI 53.0–53.6) and 18.1% (95% CI 17.9–18.4), respectively. In the heat map, the 3-year risk of rapid progression ranged from 7% in females without albuminuria, hypertension/cardiovascular disease or diabetes, to 46%–47% in males and females with severe albuminuria, diabetes and hypertension/cardiovascular disease.

**Conclusion:**

This population-based study shows that CKD stage G3 is associated with considerable morbidity in a community-based setting and underscores the need for optimized prophylactic interventions among such patients. Moreover, our data highlight the potential of using easily accessible markers in routine clinical care to identify individuals who are at high risk of rapid progression.

## Graphical Abstract

**Graphical Abstract:**
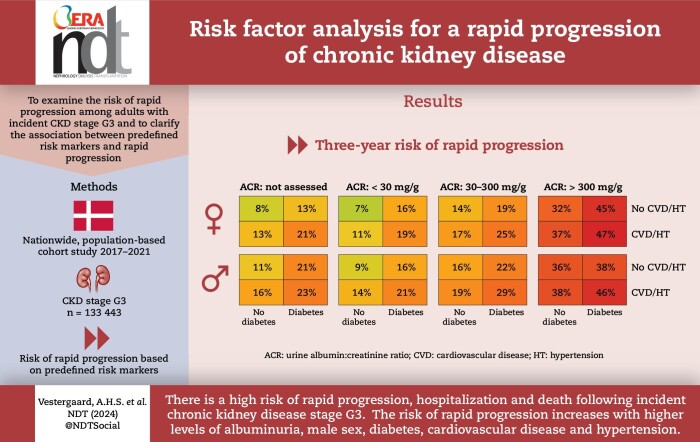

KEY LEARNING POINTS **What was known:**

- Chronic kidney disease (CKD) places a growing burden on healthcare resources worldwide due to its association with progression to kidney failure, hospitalizations and premature death.
- Contemporary data on identifying individuals in routine clinical care with new-onset CKD at high risk of rapid progression of the disease is needed to guide allocation of prophylactic interventions.

**This study adds:**

- The present population-based study shows that after 3 years, 15% of individuals with incident CKD stage G3 experienced rapid progression, 0.3% developed kidney failure, 53% were hospitalized and 18% died.
- Easily accessible markers in routine clinical care could help identify individuals at high 3-year risk of rapid CKD progression, which primarily increases with higher stage of albuminuria, and is generally higher in males than in females and in those with pre-existing diabetes, hypertension and cardiovascular disease.

**Potential impact:**

- Mild to moderate CKD is associated with considerable morbidity in a community-based setting, emphasizing the need for optimized prophylactic interventions among these patients.
- The use of easily accessible markers in routine clinical care to identify individuals who are at high risk of rapid progression of CKD may facilitate intensified interventions to prevent complications.

## INTRODUCTION

Chronic kidney disease (CKD) is a growing global health concern, largely attributed to population ageing and the increasing prevalence of metabolic and cardiovascular diseases (CVD), such as diabetes, obesity, hypertension and heart failure, which are important risk factors for CKD [1–3]. CKD now affects around 10% of adults worldwide, imposing a substantial strain on healthcare resources due to its association with progression to kidney failure, cardiovascular events, hospitalizations and premature death [3–9].

CKD stage G3 involves mild to moderate impairment of the kidneys, defined by a glomerular filtration rate (GFR) ranging from 30 to 59 mL/min/1.73 m^2^, and is generally considered irreversible [10]. Nonetheless, treatment and monitoring interventions can slow or halt progression of CKD, reducing the risk of complications [3, 11]. However, establishing clinical guidelines is challenging due to the large heterogeneity of individuals with CKD [12–14].

Among individuals with mild to moderate CKD in a community-based setting, little is known about the absolute risk of experiencing a rapid decline in GFR. Furthermore, identification of individuals at high risk of rapid progression in routine clinical care remains poorly understood. The available evidence on risk markers associated with rapid progression of CKD from stage G3 is mainly based on individuals who have received treatment in a hospital setting, making it difficult to accurately identify individuals at risk of rapidly progressing CKD in primary care [14–18]. Furthermore, most studies have focused on kidney failure, which is an outcome that may occur many years after the first signs of mild kidney impairment, if ever [14–20]. However, rapid progression increases not only the risk of kidney failure, but also the risk of other severe outcomes, such as cardiovascular events or death [18].

Therefore, it is crucial to gain further insight into the risk of rapid progression in individuals with mild to moderate CKD, and to identify individuals at high risk of rapid progression by risk markers that are easily accessible in routine clinical care. This information is imperative to identify individuals in the community setting who could potentially benefit from prophylactic interventions [21].

To address this, we conducted a nationwide, population-based cohort study among adults with incident CKD stage G3 to examine the risk of rapid progression, kidney failure, all-cause hospitalization and death. Additionally, we examined the association between predefined potential risk markers and rapid progression of incident CKD from stage G3.

## MATERIALS AND METHODS

### Study design and setting

We conducted this nationwide, population-based cohort study in Denmark (population ∼5.8 million) and included individuals with incident CKD stage G3 from 2017 through 2020 [22].

The Danish National Health Service provides universal access to tax-financed healthcare for the entire Danish population, encompassing healthcare services provided in hospitals or in primary care and partial reimbursement of prescription medicines [23].

We obtained individual-level data from national medical databases in Denmark by using the unique civil personal register (CPR) number assigned to all Danish citizens by the Danish Civil Registration system (CRS) at birth or immigration, enabling accurate and unambiguous linkage of information across different Danish registries [24, 25].

The study was registered at Aarhus University (record number 2016-051-000001/812). Under Danish law, patient consents and approvals from ethics committees are not required for non-interventional studies.

### Incident chronic kidney disease

We used the Register of Laboratory Results for Research (RLRR) to identify adult individuals (≥18 years) with biochemically defined incident CKD stage G3. The RLRR compiles laboratory data from all blood samples performed across the entire Danish population in both in- and outpatient settings and analysed at local hospital laboratories [26, 27]. Individuals with incident CKD stage G3 were identified based on estimated GFR (eGFR) values calculated from plasma creatinine measurements using the 2009 CKD Epidemiology Collaboration Creatinine equation [28]. We included blood samples with plasma creatinine measurements from the outpatient hospital setting and from primary care, including general practitioners and practicing medical specialists in 2017–2021. Based on The Kidney Disease: Improving Global Outcomes (KDIGO) criteria, individuals with incident CKD stage G3 were identified by two or more eGFR values between 30 and 59 mL/min/1.73 m^2^ separated by ≥90 days [10]. We excluded plasma creatinine measurements performed during episodes of acute kidney injury, as defined by the KDIGO criteria as an increase in plasma creatinine of ≥26.5 µmol/L within 48 h or a relative increase of at least 50% from baseline [29]. Individuals with prevalent CKD during the 3 years leading up to the study period were excluded. Due to differences in historical laboratory data availability in the five Danish geographical regions and to ensure ≥3 years of complete lookback, individuals were enrolled from 1 January 2017 in the Central and the North Denmark Region, from 1 February 2017 in the Zealand Region, from 1 October 2017 in the Capital Region and from 1 October 2018 in the Region of Southern Denmark [27].

Furthermore, we excluded individuals who had undergone kidney replacement therapy, including sustained dialysis and kidney transplantation, prior to the time of incident CKD. Information on these procedures were obtained from the Danish National Patient Registry (DNPR), a nationwide registry encompassing information from all in- and outpatient hospital contacts since 1995 [30].

### Rapid progression, kidney failure, hospitalization and mortality

Based on the KDIGO 2012 CKD guidelines, we defined rapid progression by an eGFR measurement, *m_1_*, during follow-up that was ≥5 mL/min/1.73 m^2^ lower than the eGFR at inclusion (i.e. the last of the eGFR measurements used to define incident CKD), and with an eGFR slope corresponding to an annual decline in eGFR of ≥5 mL/min/1.73 m^2^/year. The slope was estimated using a linear model, encompassing *m_1_* and all measurements taken within the preceding year. To reduce variability, we required three or more measurements with ≥90 days between the first measurement and *m_1_*. Furthermore, *m_1_* had to be confirmed by a subsequent eGFR measurement taken 30–180 days later, which should either be less than or equal to *m_1_*, or itself satisfy the criteria listed above. The date of the first confirmatory eGFR taken at day 30–180 after *m_1_* was considered the date of rapid progression.

According to the KDIGO 2012 CKD guidelines, kidney failure was defined by two or more eGFR measurements below 15 mL/min/1.73 m^2^ separated by ≥90 days in the RLRR and/or kidney replacement therapy registered in the DNPR.

Information on all-cause hospitalization, i.e. inpatient admissions, and all-cause mortality was ascertained through linkage to the DNPR and the CRS, respectively.

### Additional outcome definitions

In sensitivity analyses, we used alternative outcome definitions of CKD progression. These included: (i) potential rapid progression defined as the aforementioned rapid progression but without requiring a confirmatory subsequent eGFR measurement after 30–180 days from *m_1_*; and (ii) drop in GFR category, adopted from the KDIGO 2012 CKD guidelines defined as a drop of ≥25% in eGFR from the eGFR measurement at inclusion, concurrent with a drop in GFR category between G3a (eGFR 45–59 mL/min/1.73 m^2^), G3b (eGFR 30–44 mL/min/1.73 m^2^), G4 (eGFR 15–29 mL/min/1.73 m^2^) and G5 (eGFR <15 mL/min/1.73 m^2^).

### Covariates

We considered various risk markers associated with rapid progression, including sex, urine albumin–creatinine ratio (uACR), pre-existing diabetes, hypertension and CVD. These risk markers were chosen based on existing literature and expert consensus [14, 16, 31, 32]. In addition, several covariates were included in the study to enable characterization of the study population. Information on sex, age, geographical region of residence and marriage/cohabitation status at the time of incident CKD was obtained from the CRS. We obtained information on biomarkers from the RLRR, including plasma creatinine values at the time of incident CKD and the most recent measurements of uACR, haemoglobin, albumin and albuminuria within the preceding year. Based on the KDIGO criteria, we divided uACR values into stages of albuminuria: A1 (<30 mg/g), A2 (30–300 mg/g) and A3 (>300 mg/g) [10]. Additionally, we obtained data regarding the testing frequency of creatinine and uACR within the year preceding incident CKD. Furthermore, we stratified CKD into stage G3a and G3b. Stage G3b was defined as having two or more eGFR values of 30–45 mL/min/1.73 m^2^ measured ≥90 days apart, and stage G3a encompassed the remaining individuals. The DNPR was used to retrieve information from inpatient and outpatient hospital contacts leading to diagnoses of alcohol-related disorders and CVD, including heart failure, stroke and acute coronary syndrome, within 10 years preceding incident CKD. Smoking status was determined from surrogate markers, including hospital contacts leading to diagnoses of smoking or chronic obstructive pulmonary disease within 10 years preceding incident CKD and/or from redemption of prescription medicine associated with smoking within the year preceding incident CKD. Hypertension was identified from hospital diagnoses within 10 years preceding incident CKD and/or from redemption of two or more types of antihypertensive medicine within the preceding year. Similarly, diabetes was identified from hospital diagnoses within 10 years preceding incident CKD and/or from any redemption of antidiabetic medicine within the preceding year. Data on prescription medicine was obtained from the Danish National Prescription Registry, which contains individual-level information on all prescriptions dispensed at Danish pharmacies since 1995 [33]. This registry was also used to obtain information on use of prescribed analgesics, sodium-glucose cotransporter-2 (SGLT-2) inhibitors, renin–angiotensin–aldosterone system (RAAS) inhibitors, diuretics and statins within the year preceding incident CKD [33].

### Statistical analyses

Individuals with incident CKD stage G3 were described according to the aforementioned demographic and clinical characteristics. They were followed from the date of incident CKD until death, emigration or end of the study period (31 December 2021), whichever occurred first. During follow-up, we recorded the time of first CKD progression (rapid progression, kidney failure, potential rapid progression and drop in GFR category, separately) and hospitalization. To include death as a competing risk for non-fatal outcomes, we used the Aalen–Johansen estimator to estimate and plot the risk (cumulative incidence) of each outcome against time. We also estimated age-stratified risk of rapid progression, risk of kidney failure, all-cause hospitalization risk and all-cause mortality risk.

We developed heat maps visualizing the estimated 1-year and 3-year risk of rapid progression based on combinations of the predefined risk markers, including albuminuria stage, sex, pre-existing diabetes and/or hypertension combined with CVD.

In a supplemental analysis, we described characteristics for individuals experiencing potential rapid progression, i.e. without requiring subsequent confirmation of the lowered eGFR. Additionally, we described characteristics of individuals according to whether uACR was assessed or not. Furthermore, all analyses were rerun, applying the 2021 CKD Epidemiology Collaboration Creatinine equation.

All analyses were generated on a secure remote server hosted by The Danish Health Data Authority using SAS software version 9.4 (SAS Institute Inc., Cary, NC, USA) and R Statistical Software version 4.0.4 [34], including data.table [35] and Tidyverse [36] packages.

## RESULTS

We included 133 443 individuals with incident CKD stage G3. The median age at inclusion was 75 years [1st–3rd quartile (Q1–Q3) 69–82], and there was a large prevalence of pre-existing diabetes (18%), hypertension (62%) and CVD (21%) (Table 1). The median eGFR at inclusion was 56 mL/min/1.73 m^2^ (Q1–Q3 51–58), and the median number of eGFR measurements in the prior year was 2 (Q1–Q3 1–5) (Table 1). More than 75% of individuals with potential rapid progression had additional eGFR measurements taken after 30–180 days (Supplementary data, Table S1).

**Table 1:** Demographic and clinical characteristics of individuals with incident CKD stage G3.

Characteristics	CKD stage G3 cohort
CKD stage, *n* (%)	
G3a	128 712 (96)
G3b	4731 (4)
Calendar year of CKD, *n* (%)	
2017	25 131 (19)
2018	36 282 (27)
2019	37 347 (28)
2020	34 683 (26)
Female sex, *n* (%)	73 759 (55)
Age (years), median (Q1–Q3)	75 (69–82)
Age group (years), *n* (%)	
18–49	2675 (2)
50–59	8598 (6)
60–69	26 619 (20)
70–79	54 873 (41)
80–89	33 546 (25)
90+	7132 (5)
Living with a partner (marriage/cohabitation), *n* (%)^a^	68 971 (52)
Geographical region of residence, *n* (%)^b^	
Capital Region	39 488 (30)
Zealand Region	24 599 (18)
Region of Southern Denmark	21 819 (16)
North Denmark Region	16 924 (13)
Central Denmark Region	30 613 (23)
Comorbidities, *n* (%)	
Diabetes	24 480 (18)
Hypertension	82 553 (62)
CVD (heart failure, stroke or acute coronary syndrome)	28 399 (21)
Smoking, *n* (%)	4600 (30)
Alcohol-related disorders, *n* (%)	4996 (4)
Biomarkers, median (Q1–Q3)	
eGFR (mL/min/1.73 m^2^/year)	56 (51–58)
uACR (mg/g)	14 (7–43)
Missing, *n* (%)	89 317 (67)
Haemoglobin (mmol/L)	8 (8–9)
Missing, *n* (%)	15 804 (12)
Albumin (g/L)	38 (35–41)
Missing, *n* (%)	50 231 (38)
Albuminuria, *n* (%)	
Stage A1 (<30 mg/g)	29 897 (22)
Stage A2 (30–300 mg/g)	11 281 (8)
Stage A3 (>300 mg/g)	2880 (2)
Testing frequency in the year leading up to incident CKD, median (Q1–Q3)	
Testing frequency eGFR	2 (1–5)
Testing frequency uACR	0 (0–1)
Use of prescription medicine, *n* (%)	
Paracetamol	62 307 (47)
NSAIDs (incl. ASA)	25 365 (19)
Opioids	29 274 (22)
SGLT-2 inhibitors	4275 (3)
RAAS inhibitors	74 511 (56)
Diuretics	54 507 (41)
Statins	56 389 (42)

a Approximately 2.5 million individuals in Denmark were living with a partner in 2021 (54% of the adult population).

b Adult population in 2021: Capital Region: *n* = 1 488 231; Zealand Region: *n* = 676 086; Region of Southern Denmark: *n* = 983 395; North Denmark Region; *n* = 478 262; Central Denmark Region: *n* = 1 061 076.

NSAIDs: non-steroidal anti-inflammatory drugs; ASA: acetylsalicylic acid.

In supplemental analyses, applying the 2021 CKD Epidemiology Collaboration Creatinine equation reduced the number of individuals with incident CKD stage G3 to 105 383, encompassing individuals at a higher age, and with higher prevalence of comorbidities such as diabetes and hypertension (Supplementary data, Table S2).

### Rapid progression, kidney failure, hospitalization and mortality

**Figure 1:**
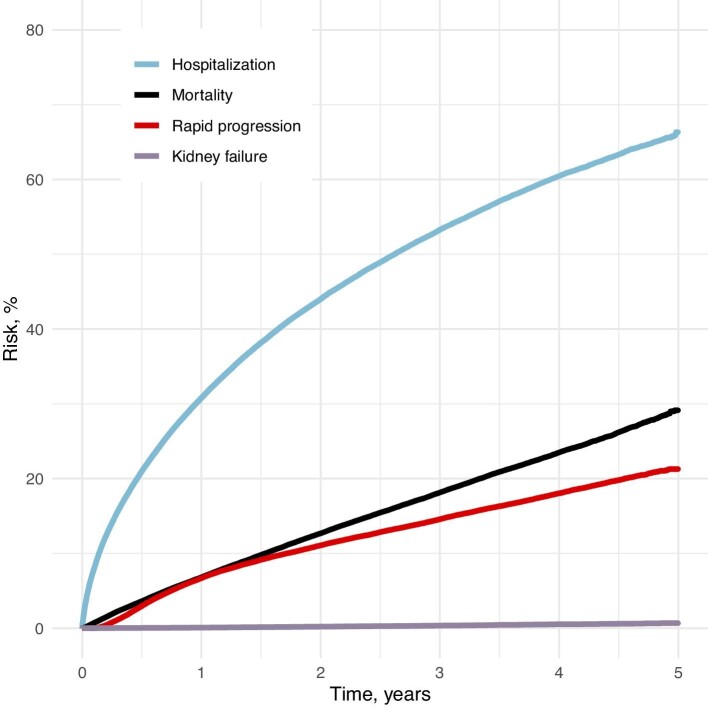
Risk of rapid progression, kidney failure, hospitalization and mortality against time.

The estimated 3-year risk of rapid progression was 14.6% [95% confidence interval (CI) 14.4–14.8]. The estimated 3-year risk of kidney failure was 0.3% (95% CI 0.3–0.4), while the all-cause hospitalization risk was 53.3% (95% CI 53.0–53.6), and the all-cause mortality risk was 18.1% (95% CI 17.9–18.4) (Fig. 1, Supplementary data, Table S3).

In the age-stratified analyses, we found the highest estimated risk of rapid progression in the youngest age group (18–49 years) and the lowest in the oldest age group (90+ years). Risk trajectories were similar among individuals aged 50–89 years. The risk of kidney failure was generally low, except for those under 50 years old, while the hospitalization risk was highest among those above 80 years old and lowest in the 50–59 years age group (Supplementary data, Fig. S1 and Table S4).

Supplemental analyses, applying the 2021 CKD Epidemiology Collaboration Creatinine equation, showed similar risk patterns for rapid progression, kidney failure, hospitalization and death (Supplementary data, Fig. S2).

### Additional outcome definitions

The estimated 3-year risk of potential rapid progression was 29.5% (95% CI 29.2–29.8), while the risk of a drop in GFR category was 14.3% (95% CI 14.0–14.5) (Supplementary data, Table S3 and Fig. S3).

### Risk markers for rapid progression

**Figure 2:**
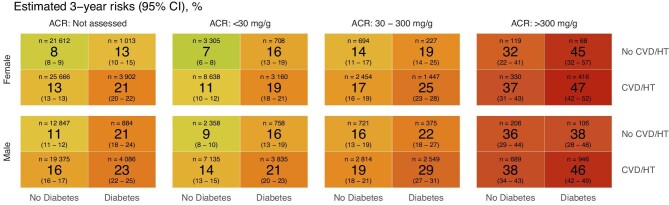
Estimated 3-year risks of rapid progression by relevant risk markers.

The estimated 3-year risk of rapid progression increased notably with higher stages of albuminuria. Moreover, the risks were generally higher in males than in females and in individuals with pre-existing diabetes and/or hypertension/CVD (Fig. 2, Supplementary data, Fig. S4). The heat map in Fig. 2 illustrates the estimated 3-year risk of rapid progression, which ranged from 7% (95% CI 6–8) in females with no albuminuria and neither diabetes nor hypertension/CVD to 47% (95% CI 41–52) in females to 46% (95% CI 42–49) in males with severe albuminuria and both diabetes and hypertension/CVD.

Similar results were found when applying the 2021 CKD Epidemiology Collaboration Creatinine equation (Supplementary data, Fig. S5).

### Urine albumin–creatinine ratio before inclusion

Within the year preceding incident CKD stage G3, 67% of the individuals did not have uACR assessed. Individuals who underwent uACR assessment were generally younger, had a higher prevalence of hypertension or diabetes and were more often prescribed SGLT-2 inhibitors, RAAS inhibitors, diuretics or statins (Supplementary data, Table S5).

## DISCUSSION

This nationwide cohort study used unique population-based laboratory data on plasma creatinine measurements performed in Denmark in 2017–21. Among individuals with incident CKD stage G3, the study revealed that after 3 years, 15% had experienced rapid progression, 53% had been hospitalized and 18% had died, while the risk of kidney failure was found to be as low as 0.3%. Furthermore, our findings suggest that the risk of rapid progression increases with higher stage of albuminuria, and that the risk is generally higher in males than in females and in individuals with pre-existing diabetes and/or hypertension/CVD. Among individuals with severe albuminuria, diabetes and hypertension/CVD, nearly half experienced rapid progression within 3 years.

Most previous studies of CKD progression have focused on kidney failure as the only clinical outcome [14–20]. While this outcome is indeed relevant, these studies often focus on long-term risks and may not adequately address changes in GFR that are important for early identification of individuals at high risk of rapid progression and severe complications [14–20]. Early identification and treatment initiation play a vital role in delaying or preventing the progression to kidney failure and other complications, which is important from a clinical and political perspective [3, 11]. Our study provides novel insights into the risk of rapid progression in individuals with mild to moderate CKD and associated risk markers. Notably, there is a scarcity of previous community-based research on the risk of rapid progression, as defined by the KDIGO criteria, kidney failure, hospitalization and death [4, 20, 37]. Among 35 195 individuals with incident CKD stage G3, Go *et al*. (2018) estimated a 2-year risk of rapid progression of 18.1% (95% CI 17.7–18.5) when defined by a decline in eGFR of ≥4 mL/min/1.73 m^2^/year [37]. This definition corresponded largely with our sensitivity analysis definition of potential rapid progression, yielding a 3-year risk of 29.5% (95% CI 29.2–29.8) [37]. In 2004, Go *et al*. examined the association between eGFR and the risk of all-cause hospitalization and death in a cohort of 1 120 295 adults from the general population in the USA [4]. Although our estimates cannot be directly compared, they also reported a substantial risk of all-cause hospitalization and death following incident CKD with eGFR values between 45–59 and 30–44 mL/min/1.73 m^2^/year [4]. Our findings also align with those of Eriksen *et al*. (2006), examining 6863 individuals with incident CKD stage G3 (defined by the KDIGO criteria) [20]. Their estimated 5-year risk of kidney failure risk and death was 2% (95% CI 1–2) and 32% (95% CI 30–34), respectively [20].

In the present study, the risk of rapid progression increased with higher stage of albuminuria, a finding corroborated in previous studies [13, 38–40]. Similarly, males have been reported to have higher risk of kidney failure and decline in kidney function than females [13, 20]. Population-based studies have also established that individuals with diabetes have higher risk of kidney failure, in line with our findings on rapid progression [13, 18]. Hypertension is a well-known consequence of declining kidney function but also an important upstream factor in CKD development and progression [13]. Our findings are in line with previous studies that have reported a considerable risk of death and progression to kidney failure associated with the presence of hypertension/CVD in addition to CKD. While the covariates examined in the present study do not encompass all potential risk markers for rapid progression, they serve as valuable risk stratification tools due to their widespread availability in most cohorts. The study included commonly suspected risk markers for rapid progression, underscoring the importance of albuminuria, even in individuals with CKD stage G3 without diabetes.

The present study has several strengths, including its nationwide, unselected, population-based design in a healthcare system with universal coverage. This enabled individual-level linkage between national medical databases with prospectively collected data, generally considered highly complete and valid [26, 27, 33, 41]. Additionally, a major strength of the study is the inclusion of a confirmatory eGFR measurement in defining rapid progression. This measurement was performed in over 75% of the individuals with potential rapid progression and there were no considerable differences in characteristics between those who had an additional eGFR measurement and those who did not.

Even so, some limitations should be considered. The study relied on clinical data, which means that individuals had an indication for plasma creatinine measurements in everyday clinical care. While eGFR is routinely measured in a community setting, uACR is not. In our study dataset of the general population, we observed 66 million plasma creatinine measurements compared with only 5.1 million uACR measurements (data not shown). We could not, therefore, include uACR measurements in the definition of incident CKD, leading to a likely underestimation of the occurrence of CKD in the general population in Denmark.

However, as any standard blood test panel in Denmark typically includes a creatinine measurement, we assume that the risk of selection bias is minor, since individuals with incident CKD stage G3 were identified from complete data on all blood samples performed during any consultations in primary care and in outpatient hospital clinics.

Although the number of hospitals covered in the RLRR increased throughout the study period, any incompleteness is likely only due to technical factors related to data reporting to the registries from the hospital or not, rather than selection of individuals [27]. We minimized the risk of including individuals with pre-existing prevalent CKD by requiring ≥3 years of complete lookback.

The definition of rapid CKD progression used in the present study, derived from the KDIGO guidelines, may be considered somewhat arbitrary. Therefore, we performed a sensitivity analysis using an alternative outcome definition (drop in GFR category). Notably, this alternative analysis exhibited a similar pattern to the main analysis.

While the definitions of covariates employed in this study were generally considered robust and valid [42–44], some variables like alcohol-related disorders and smoking status were only accessible by surrogate markers, including hospital diagnoses and redemption of prescription medicine, which may have led to misclassification.

Consistent with standard clinical practice, a small proportion of individuals underwent uACR assessment in the year before incident CKD stage G3. Individuals who did undergo uACR assessment generally had poorer overall health, e.g. diabetes and hypertension, which may have led clinicians to consider them at a higher risk of albuminuria. Individuals without uACR assessment and individuals having uACR <30 mg/g had a similar risk of rapid progression, indicating that most individuals without uACR assessment did not have albuminuria. However, it is crucial to emphasize that the lower risks observed in the absence of uACR assessment should not be interpreted as a suggestion to forgo uACR assessments in individuals with or at risk of CKD.

In conclusion, we found a substantial risk of rapidly progressing CKD, hospitalization and death in the years following incident CKD stage G3, while the risk of kidney failure was low. The risk of rapid progression increased markedly with higher levels of albuminuria, and it was higher in males than in females and in those with pre-existing diabetes and/or hypertension/CVD.

The present population-based study highlights the potential for using readily available markers in routine clinical care to identify individuals at particularly high risk of rapid progression of CKD who may benefit from regular monitoring and prophylactic interventions to slow further decline in kidney function and associated adverse events.

## Supplementary Material

gfad271_Supplemental_File

## Data Availability

The data supporting the findings of this study can be obtained from Statistics Denmark, the central authority for Danish statistics.
