# Supplementary material for: Risk factor analysis for a rapid progression of chronic kidney disease
Source: Nephrol Dial Transplant. 2024 Jan 2;39(7):1150–8. doi: 10.1093/ndt/gfad271 (PMC11210987; doi:10.1093/ndt/gfad271)
Supplement: gfad271_Supplemental_File [file gfad271_supplemental_file.pdf]

Supplementary Table S1 Demographic and clinical characteristics of individuals with *potential* rapid progression according to whether they had additional eGFR measurements taken after 30-180 days.

Characteristics	Additional eGFR measured	No additional eGFR measured
Total	29,037 (100)	9,042 (100)
Female sex, n (%)	14,282 (49)	4,878 (54)
Age, median (Q1-Q3)	75 (69-80)	77 (71-83)
Age group, n (%)		
18-49	749 (3)	133 (1)
50-59	1,778 (6)	413 (5)
60-69	6,057 (21)	1,503 (17)
70-79	12,709 (44)	3,694 (41)
80-89	6,927 (24)	2,730 (30)
90+	817 (3)	569 (6)
Comorbidities, n (%)		
Diabetes	7,908 (27)	1,967 (22)
Hypertension	20,717 (71)	6,169 (68)
Cardiovascular disease (heart failure, stroke or acute coronary syndrome)	21,590 (74)	6,478 (72)
Biomarkers, median (Q1-Q3)		
Urine albumin-creatinine ratio (mg/g)	22 (9-84)	18 (8-59)
Missing, n (%)	18,045 (62)	5,983 (66)
Albuminuria, n (%)		
Stage A1 (<30 mg/g)	6,300 (22)	1,892 (21)
Stage A2 (30-300 mg/g)	3,350 (12)	891 (10)
Stage A3 (>300 mg/g)	1,342 (5)	276 (3)
Follow-up after rapid progression, n (%)		
Followed for 180 days	26,030 (90)	5,612 (62)
Censored within 30-180 days	1,906 (7)	1,643 (18)
Died within 30-180 days	1,101 (4)	837 (9)
Censored in < 30 days	-	533 (6)
Died in < 30 days	-	417 (5)

Abbreviations: CKD, chronic kidney disease; eGFR, estimated glomerular filtration rate; Q1-Q3: quartile 1-3.

Supplementary Table S2 CKD-EPI 2021 equation: Demographic and clinical characteristics of individuals with incident CKD stage G3.

Characteristics	CKD stage G3 cohort
CKD stage, n (%)	
G3a	101,729 (97)
G3b	3,654 (3)
Calendar year of CKD, n (%)	
2017	19,824 (19)
2018	28,278 (27)
2019	29,531 (28)
2020	27,750 (26)
Female sex, n (%)	57,953 (55)
Age, median (Q1-Q3)	76 (70 - 83)
Age group (years), n (%)	
18-49	2,026 (2)
50-59	5,808 (6)
60-69	18,295 (17)
70-79	42,210 (40)
80-89	30,054 (29)
90+	6,990 (7)
Living with a partner (marriage/cohabitation), n (%)[1]	52,284 (50)
Geographical region of residence, n (%)[2]	
Capital Region	30,443 (29)
Zealand Region	19,526 (19)
Region of Southern Denmark	17,271 (16)
North Denmark Region	13,742 (13)
Central Denmark Region	24,401 (23)
Comorbidities, n (%)	
Diabetes	22,052 (21)
Hypertension	70,698 (67)
Cardiovascular disease (heart failure, stroke or acute coronary syndrome)	25,274 (24)
Smoking, n (%)	33,480 (32)
Alcohol-related disorders, n (%)	4,020 (4)
Biomarkers, median (Q1-Q3)	
eGFR (ml/min/1.73 m^2/year)	55 (51 - 58)
Urine albumin-creatinine ratio (mg/g)	17 (7 - 53)
Missing, n (%)	68,650 (65)
Haemoglobin (mmol/l)	8 (8 - 9)
Missing, n (%)	11,356 (11)
Albumin (g/l)	38 (35 - 41)
Missing, n (%)	37,226 (35)
Albuminuria, n (%)	
Stage A1 (<30 mg/g)	23,624 (22)
Stage A2 (30-300 mg/g)	10,125 (10)
Stage A3 (>300 mg/g)	2,929 (3)
Testing frequency in the year leading up to incident CKD, median (Q1-Q3)	
Testing frequency eGFR	3 (1 - 5)
Testing frequency urine albumin-creatinine ratio	0 (0 - 1)
Use of prescription medicine, n (%)	
Paracetamol	51,953 (49)
NSAIDs (incl. ASA)	19,629 (19)
Opioids	24,712 (23)
SGLT-2 inhibitors	3,770 (4)
RAAS inhibitors	63,074 (60)
Diuretics	46,792 (44)
Statins	46,984 (45)

Abbreviations: CKD, chronic kidney disease; eGFR, estimated glomerular filtration rate; NSAIDs; non-steroidal anti-inflammatory drugs; ASA: acetylsalicylic acid; Q1-Q3: quartile 1-3; SGLT-2: sodium-glucose cotransporter-2; RAAS: renin-angiotensin-aldosterone system.

[1] Approximately 2.5 million individuals in Denmark were living with a partner in 2021 (54% of the adult population).

[2] Adult population in 2021: Capital Region: n=1 488 231; Zealand Region: n=676 086; Region of Southern Denmark: n=983 395; North Denmark Region; n=478 262; Central Denmark Region: n=1 061 076.

Supplementary Table S3 Risk of CKD progression after 1 year and 3 years.

Outcome	1-year risk, % (95% CI)	3-year risk, % (95% CI)
Rapid progression	6.7 (6.6 - 6.8)	14.6 (14.4 - 14.8)
All-cause hospitalisation	30.8 (30.5 - 31.0)	53.3 (53.0 - 53.6)
All-cause mortality	6.8 (6.7 - 6.9)	18.1 (17.9 - 18.4)
Potential rapid progression	16.7 (16.5 - 16.9)	29.5 (29.2 - 29.8)
Drop in GFR category	5.4 (5.3 - 5.5)	14.3 (14.0 - 14.5)
Kidney failure	0.1 (0.1 - 0.1)	0.3 (0.3 - 0.4)

Abbreviations: CI, confidence interval.

Supplementary Table S4 Risk of rapid progression, hospitalisation and mortality after 1 year and 3 years by age groups.

	Rapid progression		All-cause hospitalisation		All-cause mortality	
Age group (years)	1-year risk, % (95% CI)	3-year risk, % (95% CI)	1-year risk, % (95% CI)	3-year risk, % (95% CI)	1-year risk, % (95% CI)	3-year risk, % (95% CI)
18-49	10.6 (9.5 - 11.8)	21.2 (19.5 - 22.9)	29.9 (28.2 - 31.6)	48.8 (46.7 - 50.9)	2.1 (1.6 - 2.7)	4.8 (4.0 - 5.8)
50-59	6.4 (5.9 - 6.9)	13.1 (12.4 - 13.9)	27.6 (26.7 - 28.5)	44.7 (43.5 - 45.9)	3.2 (2.9 - 3.6)	7.2 (6.6 - 7.9)
60-69	7.1 (6.8 - 7.4)	14.7 (14.2 - 15.2)	27.8 (27.2 - 28.3)	46.7 (46.0 - 47.4)	4.0 (3.8 - 4.3)	10.0 (9.6 - 10.4)
70-79	6.9 (6.7 - 7.1)	15.4 (15.1 - 15.8)	29.5 (29.1 - 29.9)	51.7 (51.3 - 52.2)	5.0 (4.8 - 5.2)	13.4 (13.0 - 13.7)
80-89	6.3 (6.1 - 6.6)	14.3 (13.9 - 14.7)	34.4 (33.9 - 34.9)	60.9 (60.3 - 61.5)	9.9 (9.6 - 10.2)	27.8 (27.2 - 28.3)
90+	3.9 (3.5 - 4.4)	8.1 (7.4 - 8.8)	38.7 (37.5 - 39.8)	64.5 (63.3 - 65.7)	22.2 (21.2 - 23.2)	55.7 (54.3 - 57.0)

Supplementary Table S5 Demographic and clinical characteristics of individuals with CKD stage G3 according to whether albumin-creatinine ratio was assessed at inclusion.

Characteristics	ACR assessed	ACR not assessed
CKD stage, n (%)		
G3a	42,844 (97)	85,868 (96)
G3b	1,282 (3)	3,449 (4)
Calendar year of CKD, n (%)		
2017	7,516 (17)	17,615 (20)
2018	11,266 (26)	25,016 (28)
2019	12,579 (29)	24,768 (28)
2020	12,765 (29)	21,918 (25)
Female sex, n (%)	21,619 (49)	52,140 (58)
Age, median (Q1-Q3)	74 (68 - 80)	76 (69 - 82)
Age group, n (%)		
18-49	977 (2)	1,698 (2)
50-59	2,846 (6)	5,752 (6)
60-69	9,524 (22)	17,095 (19)
70-79	19,701 (45)	35,172 (39)
80-89	9,901 (22)	23,645 (26)
90+	1,177 (3)	5,955 (7)
Living with a partner (marriage/cohabitation), n (%)[1]	24,639 (56)	44,332 (50)
Geographical region of residence, n (%)[2]		
Capital Region	10,286 (23)	29,202 (33)
Zealand Region	7,234 (16)	17,365 (19)
Region of Southern Denmark	8,664 (20)	13,155 (15)
North Denmark Region	5,133 (12)	11,791 (13)
Central Denmark Region	12,809 (29)	17,804 (20)
Comorbidities, n (%)		
Diabetes	14,606 (33)	9,874 (11)
Hypertension	33,324 (76)	49,229 (55)
Cardiovascular disease (heart failure, stroke or acute coronary syndrome)	9,169 (21)	19,230 (22)
Smoking, n (%)	12,897 (29)	27,703 (31)
Alcohol-related disorders, n (%)	1,374 (3)	3,622 (4)
Biomarkers, median (Q1-Q3)		
eGFR (ml/min/1.73 m^2/year)	56 (52 - 58)	55 (51 - 58)
Haemoglobin (mmol/l)	8 (8 - 9)	8 (8 - 9)
Missing, n (%)	6,750 (15)	9,054 (10)
Albumin (g/l)	39 (36 - 42)	38 (35 - 41)
Missing, n (%)	17,308 (39)	32,923 (37)
Testing frequency in the year leading up to incident CKD, median (Q1-Q3)		
Testing frequency eGFR	3 (1 - 5)	2 (1 - 5)
Use of prescription medicine, n (%)		
Paracetamol	19,699 (45)	42,608 (48)
NSAIDs (incl. ASA)	7,857 (18)	17,508 (20)
Opioids	8,296 (19)	20,978 (23)
SGLT-2 inhibitors	2,911 (7)	1,364 (2)
RAAS inhibitors	32,277 (73)	42,234 (47)
Diuretics	22,474 (51)	32,033 (36)
Statins	24,743 (56)	31,646 (35)

Abbreviations: ACR, albumin-creatinine ratio; CKD, chronic kidney disease; eGFR, estimated glomerular filtration rate; NSAIDs; non-steroidal anti-inflammatory drugs; ASA: acetylsalicylic acid; Q1-Q3: quartile 1-3; SGLT-2: sodium-glucose cotransporter-2; RAAS: renin-angiotensin-aldosterone system.

Supplementary Figure S1 Age-stratified risk of rapid progression, hospitalisation and mortality.

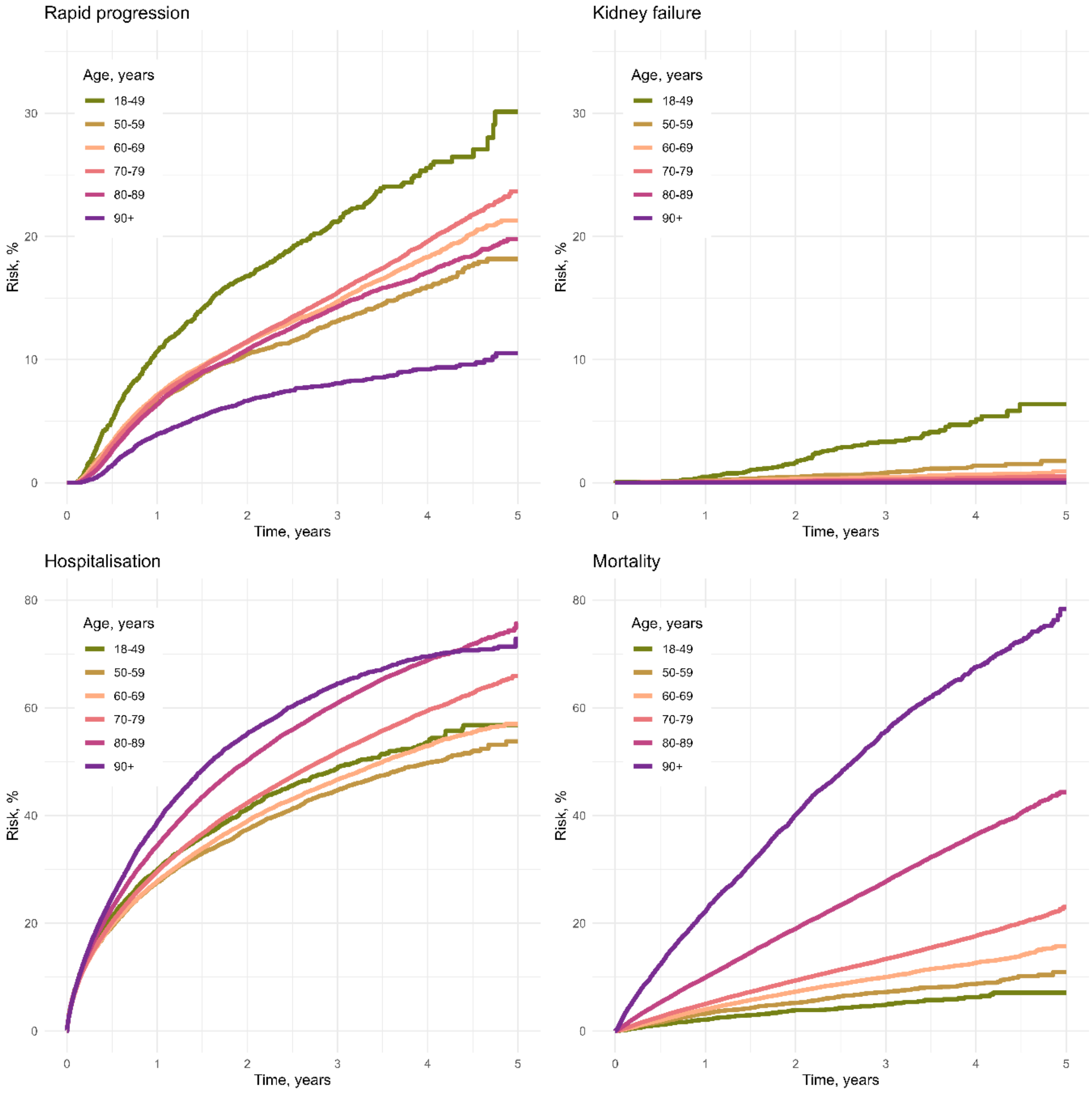

Supplementary Figure S2 CKD-EPI 2021 equation: Risk of rapid progression, kidney failure, hospitalisation and mortality against time.

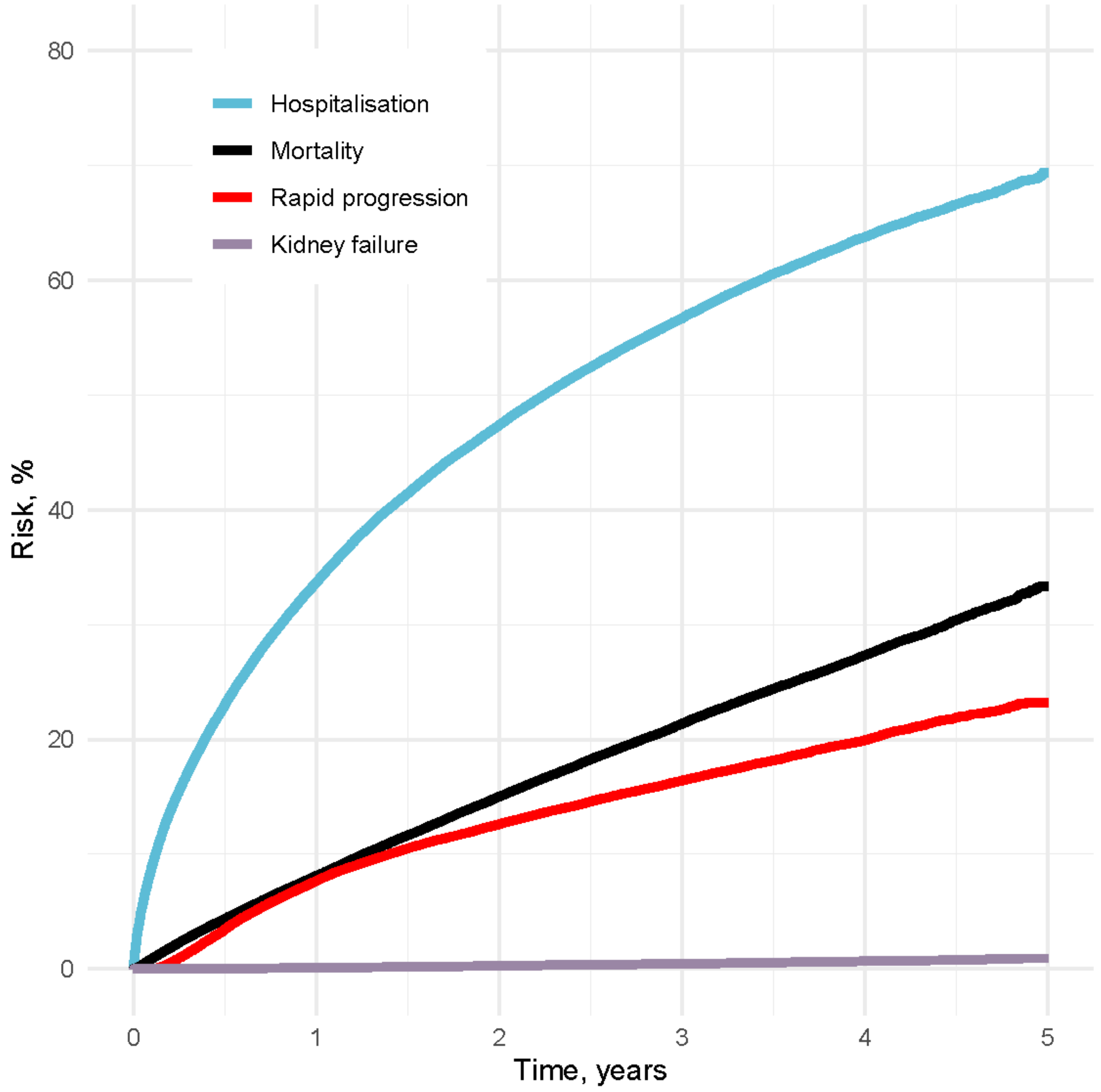

Supplementary Figure S3 Risk of *potential* rapid progression and drop in GFR category.

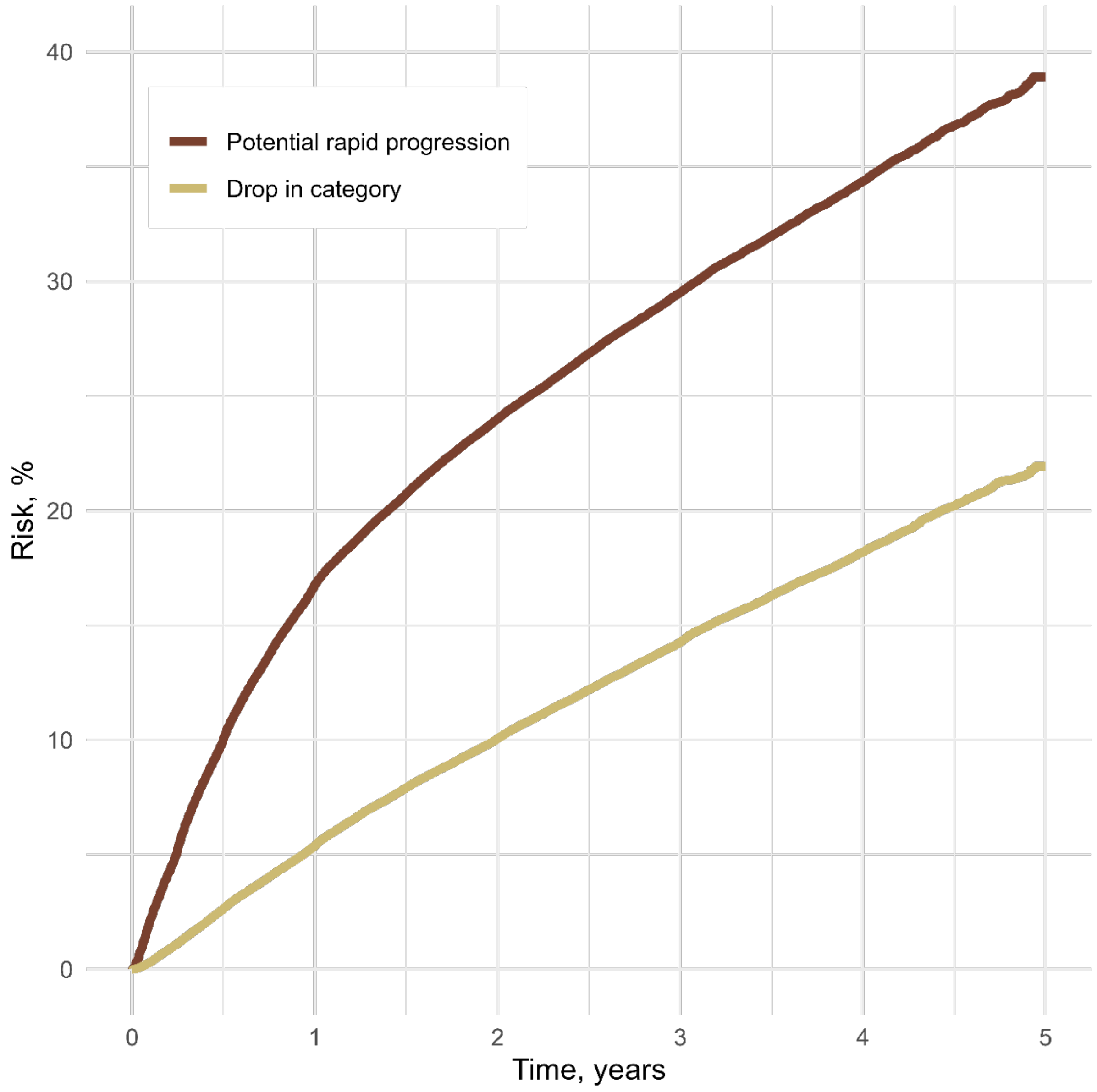

Supplementary Figure S4 Estimated 1-year risks of rapid progression by relevant markers.

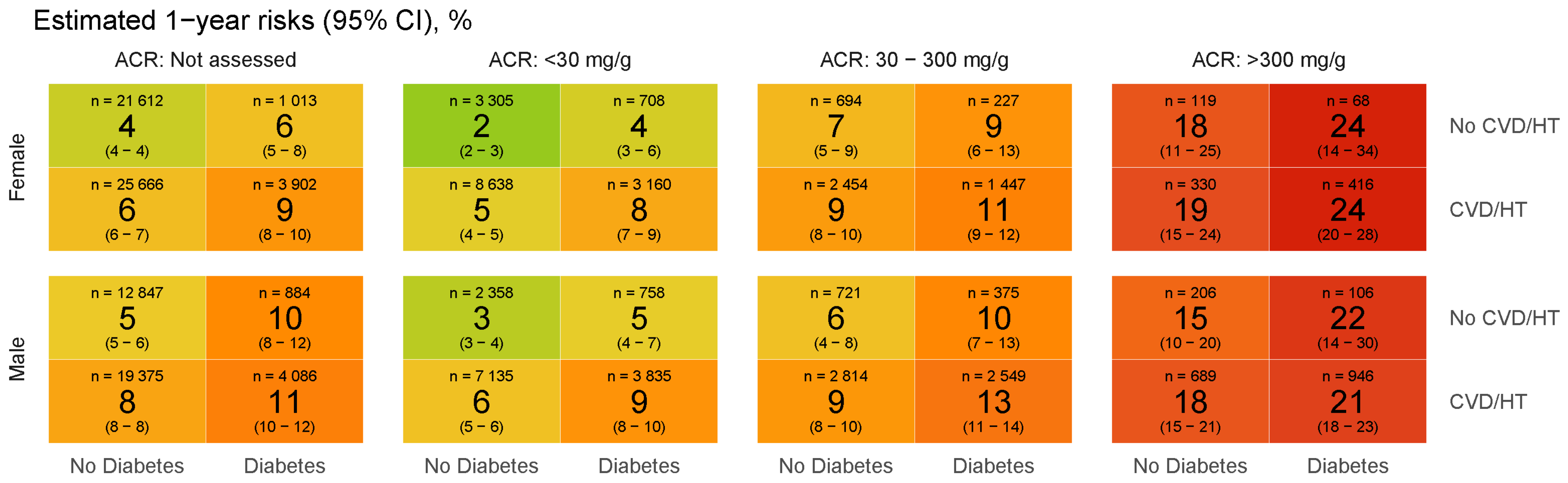

Abbreviations: CI, confidence interval; ACR, albumin-creatinine ratio; CVD, cardiovascular disease; HT, hypertension.

Supplementary Figure S5 CKD-EPI 2021 equation: Estimated 3-year risks of rapid progression by relevant markers.

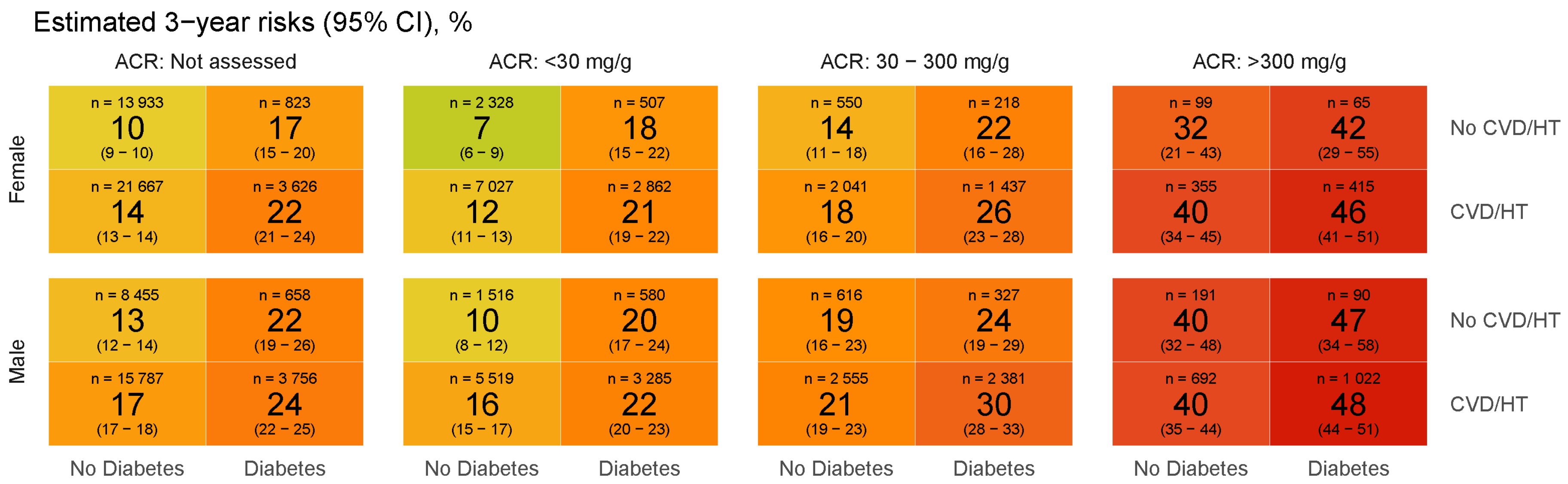

Abbreviations: CI, confidence interval; ACR, albumin-creatinine ratio; CVD, cardiovascular disease; HT, hypertension.
